# Implementation through translation: a qualitative case study of translation processes in the implementation of quality improvement collaboratives

**DOI:** 10.1186/s12913-023-09201-4

**Published:** 2023-03-13

**Authors:** Kathrine Carstensen, Anne Mette Kjeldsen, Stina Lou, Camilla Palmhøj Nielsen

**Affiliations:** 1 Public Health Research, Central Denmark Region, Department of Public Health, DEFACTUM, Aarhus University, Olof Palmes Alle 15, Aarhus N, DK-8200 Denmark; 2 Department of Political Science, Aarhus University, Bartholins Allé 7, Aarhus C, DK-8000 Denmark; 3 Public Health Research, Central Denmark Region, Department of Clinical Medicine, DEFACTUM, Aarhus University, Olof Palmes Alle 15, Aarhus N, DK-8200 Denmark

**Keywords:** Quality improvement collaboratives, Implementation, Translation, Health Care

## Abstract

**Background:**

Quality improvement collaboratives (QICs) are used extensively to implement quality improvement in healthcare, and current research is demonstrating positive yet varying evidence. To interpret the effectiveness results, it is necessary to illuminate the dynamics of QIC implementation in specific contexts. Using Scandinavian institutionalist translation theory as a theoretical framework, this study aims to make two contributions. First, we provide insights into the dynamics of the translation processes inherent in QIC implementation. Second, we discuss the implications of the translation processes as experienced by participating actors.

**Methods:**

We used empirical data from a qualitative case study investigating the implementation of QICs as an approach to quality improvement within a national Danish healthcare quality program. We included two diverse QICs to allow for exploration of the significance of organizational complexity for the translation processes. Data comprised qualitative interviews, participant observation and documentary material.

**Results:**

Translation was an inherent part of QIC implementation. Key actors at different organizational levels engaged in translation of their implementation roles, and the QIC content and methodology. They drew on different translation strategies and practices that mainly materialized as kinds of modification. The translations were motivated by deliberate, strategic, and pragmatic rationales, contingent on combinations of features of the actors’ organizational contexts, and the transformability and organizational complexity of the QICs. The findings point to a transformative power of translation, as different translations led to various regional and local QIC versions. Furthermore, the findings indicate that translation affects the outcomes of the implementation process and the QIC intervention. Translation may positively affect the institutionalization of the QICs and the creation of professional engagement and negatively influence the QIC effects.

**Conclusion:**

The findings extends the current research concerning the understanding of the dynamics of the translation processes embedded in the local implementation of QICs, and thus constitute a valuable contribution to a more sustainable and effective implementation of QICs in healthcare improvement. For researchers and practitioners, this highlights translation as an embedded part of the QIC implementation process, and encourages detailed attention to the implications of translation for both organizational institutionalization and realisation of the expected intervention outcomes.

## Background

Deficiencies in the quality of healthcare and the risk providing low-quality patient treatment continue to be of significant concern as evidence documenting gaps between actual and recommended clinical practices continues to accumulate [1–3]. As a consequence, considerable emphasis has been placed on improving healthcare through the implementation of evidence-based practices, and policymakers and healthcare leaders seek effective approaches to improve healthcare processes and, ultimately, patient outcomes [1–3]. The quality improvement collaborative model (QICs) is one approach that has become extensively used for quality improvement and implementation of evidence-based practices in healthcare [2–6]. Although different approaches exist, the best-known version is the Breakthrough Series Collaborative Model developed in the United States by the Institute for Healthcare Improvement (IHI). In this model, a QIC is a bottom-up-driven approach consisting of a learning collaborative of multidisciplinary-assembled teams of professionals from different organizational sites, that cooperate within and across teams to improve healthcare processes and outcomes for a targeted area of care [6, 7].

The scientific evidence of QICs’ effects generally shows positive, but varying results. A recent systematic review concluded that QICs were largely effective in reaching their stated aims, with 83% claiming improvement in at least one of the outcomes addressed [5, also 3, 4, 6]. However, in the QIC literature, there has only been limited attention to the processes of implementation of QICs, including how QICs work in specific settings. This challenges the interpretation of effect results in terms of the scope and boundary conditions for successful QIC implementation [2, 5, 6, 8, 9].

Recently, a small number of studies have begun to extend our insights into the nature of QIC implementation (e.g. [9–14]). Among studies focusing on factors that contribute to successful QIC implementation, Wilson et al. (2003) [10] found that critical determinants of QICs’ effects include the selection of a topic compatible with organizational needs; multidisciplinary assembled quality improvement teams (QI teams); and the support of senior organizational leaders. Dückers et al. (2009) [13] found that support from change agents, e.g., in arranging group meetings and facilitating the use of QIC methodology, positively influenced the number of changes made by QIC teams. Other studies have focused on specific parts of QIC implementation. For example, Shaw et al. (2012) [11] investigated how the QICs shaped local quality improvement efforts by offering a way of disseminating healthcare innovations through multidisciplinary collaboration and learning. These studies provide important insights regarding QIC implementation; yet they tend to treat QICs as a universal approach with predefined, fixed elements. This way, implementation is presented as a linear process in which the QIC intervention is expected to uniformly “trickle down” in and across local contexts as a one-size-fits-all approach [9, 15, 16].

Broer et al. (2010) [9] applied a more dynamic approach by investigating how the QIC content and improvement measurements developed during implementation. They found a translation of the focus of the QIC and showed how the selected quality measurements shaped the improvement initiatives and affected the actors involved. Stoopendaal and Bal (2013) [17] investigated the practices required to enable and sustain QIC improvements. They found that the QIC goals were not accomplished linearly, but rather were shaped by various translation processes and depended highly on local negotiations. With these results, the studies point to the increasing acknowledgment that intervention effects are context-dependent and that interventions during their implementation are translated to fit the local contexts [18–21]. Thus, to understand the outcomes of QICs and similar interventions, empirical research is needed that extends our understanding of the translation processes embedded in the implementation of QICs in local contexts, and their implications [4–6, 9, 14, 17, 22, 23].

Against this backdrop, the present paper aims to make two contributions. First, we provide insights into the dynamics of the translation processes inherent in QIC implementation. Second, we discuss the implications of the translation processes as experienced by participating actors. Our findings may contribute to promoting a more sustainable and effective use of QICs in healthcare improvement and thus to more effective investment in quality improvement. Theoretically, we draw on translation theory, which offers an analytical perspective to illuminate the context-specific translation processes and practices inherent in QIC implementation [24–26]. Our empirical data derives from a qualitative case study investigating the use of QICs within a Danish national program for quality in healthcare [27].

## Theoretical framework: implementation as translation

Our theoretical framework draws on Scandinavian neo-institutionalist translation theory, which was developed to advance understanding of the spread of organizational ideas, concepts, and practices [24, 25, 28, 29]. Within this theoretical perspective, translation is about transformation, as translation refers to the process “(…) *in which ideas and models are adapted to local contexts as they travel across time and space*” [24, 26]. Hence, traveling organizational ideas, such as QICs, do not simply “trickle down” in a linear way when transferred from the source context to the recipient contexts. Instead, they are reformulated and redefined every time they are implemented by organizations [16, 24, 26, 28–31]. Thus, with this theoretical framework, we understand implementation as a process of adaptation.

Translation theory is chosen as theoretical framework because of its clear emphasis on agency. Organizational actors are expected to intentionally influence the implementation of new ideas by translating them to their local organizational contexts [25, 26, 29–32]. Such an agency perspective fits the present paper’s ambition to examine the dynamics of the translation processes inherent in QIC implementation, and the implications of the translation processes as experienced by participating actors. Furthermore, the QIC approach is essentially a bottom-up driven model designed to strengthen the role of health professionals in driving quality improvements. This calls for a theoretical perspective that focuses on how the professionals implement and translate the QICs in their local context.

For the purpose of this paper, we rely on Røvik’s (2007, 2016, 2019) [25, 33, 34] conceptual framework for the analysis of the translation of organizational ideas. In contrast to other translation scholars such as Latour (1986) and Sahlin-Andersson (1996), who primarily focus on identifying the actors facilitating translations between organizations, Røvik fits the aim of the present paper by drawing attention to the organizations’ contextualization of the traveling idea and thus the intra-organizational translation practices [35]. Particularly, we draw on three key aspects of Røviks conceptual framework: (1) intra-organizational translation as a rule-based activity, (2) the contextual conditions influencing the translations, and (3) the implications of the translations for the outcome of the translated idea. Regarding the first, Røvik argues that intra-organizational translation is a rule-based activity guided by underlying *modes* and *rules* of translation. Translation *mode* refers to the specific strategy of the translation practice. It may reflect deliberate intentions, but may also be taken for granted, echoing habits, culture, and traditions in the organizational context. Translation *rule* refers to the organizational guidelines for the appropriate translation of the idea. They may be explicitly stated but are often implicit and expressed through practices [25, 33, 34]. A main argument is that some translation modes and rules lead to practically no transformation, while others can lead to profound transformations of the traveling idea [25]. Røvik distinguishes between three different ideal types of translation modes: the reproducing, the modifying, and the radical mode. Each has its own characteristic *rules* of translation. In the reproducing mode, the recipient organization attempts to replicate (*copying*) the original idea. The modifying mode is a mix of replication and adjustment. Thus, it may involve toning down or subtracting (*omission*) some elements of the translated idea, and/or supplementing the idea with additional elements (*addition*). Finally, the radical mode involves a significant transformation of the original idea (*alteration*) [25, 33]. Given the complexity of the QIC intervention, we expect to identify the use of a mix of different translation modes and rules. We empirically examine to what extent these modes and rules follow identifiable patterns as suggested by Røvik, and whether the outcomes of the local translation processes are unique or display some common features. In other words, whether translation leads to heterogeneity or homogeneity across local contexts.

Regarding the contextual conditions, translation processes, including the use of translation modes and rules, are according to Røvik influenced by (1) features of the source context, meaning the translatability of the transferred idea; (2) the transformability of the translated idea, meaning the degrees of freedom that the translators have to interpret and make their own versions of the idea; and (3) features of the recipient context and its relationship to the source context, meaning characteristics of the recipient context and the degree of similarity between the recipient and source contexts [25]. Røvik specifies how the relations between the contextual conditions and the translations are complex. Thus, translation will typically be influenced by combinations of conditions and therefore translations will typically vary across different organizational contexts. Moreover, the contextual conditions can be used both consciously and unconsciously by actors in the translation [25]. In relation to the QICs in our study, we expect that both characteristics of the QICs as translated ideas (e.g. their inherent leeway to co- or even self-determination of content and methodology) and characteristics of the regional and local contexts (e.g. managerial strategies, history and traditions, and available competencies and resources) may constitute influential contextual conditions for the intra-organizational translation practices. We empirically examine the complexity of the relations between the translations and the contextual conditions influencing them.

Finally, with respect to the implications of translation, a key point of Røvik’s framework is that translation makes a difference for the outcome of the translated idea. Thus, it may be possible to identify appropriate and less appropriate translations [25, 34]. For example, a translation can be so radical that the recipient version is hardly accepted as a translation [25]. However, Røvik does not further specify what constitutes appropriate and less appropriate translation nor how much translation is acceptable. The appropriateness of the translation thus remains an empirical question.

## Methods

The analysis was based on a qualitative case study investigating the use of QICs within a Danish national program for quality in healthcare. The study applied a single-case design with two embedded sub-cases to investigate the complex and context-dependent translation processes [36–38].

### Study setting

In 2015, the Danish government introduced a national healthcare quality program to support the continuous improvement of the entire healthcare system [27, 39]. The program introduced QICs as an improvement approach [27, 39, 40]. This political decision was based on different policy objectives. The QICs were introduced to create consistent quality improvement within clinical areas characterized by unsatisfactory quality, and as a proposed solution for bridging the quality gap between best evidence and current practice. Furthermore, an objective was to introduce a bottom-up driven quality improvement approach with local healthcare professionals setting the agendas, and driving the implementation [27]. The political decision to use QICs was fuelled by previous Danish experiences with the use of QICs and the successful use of QICs internationally. Evidence-based discussions of the effectiveness of QICs were rather absent in the decision-making processes, and the introduction of the QICs was not followed by a formal plan for a systematic evaluation of their implementation and effects [27].

Although the decision to apply QICs was made by the government and may be seen as an element in centralizing the quality improvement work in the Danish healthcare system, the five Danish regions were involved in developing the organizational set-up. A main motivation for this was to acknowledge the expertise of healthcare professionals and strengthen a bottom-up and network-based approach to quality improvement, to accommodate an increasing dissatisfaction among healthcare professionals who had experienced heavy bureaucracy through previous approaches to quality improvement [27]. The QICs largely follow the structure of IHI’s Breakthrough Series Collaboratives [27, 40]. All QICs are implemented nationally across the five Danish regions and participation is mandatory. Each region is responsible for appointing local QI teams to participate in the QIC. The QI teams consist of employees and managers representing different professional groups and, if relevant, different hospital departments/municipal organizational units, jointly responsible for the improvement work. With exceptions, a ‘typical’ QIC involves QI teams from 15 to 25 sites with 3–12 participants each. The overall topic of QICs is decided by a national steering committee while the QIC objectives, content, and measurements are defined and formulated in a project description by the national project management and a QIC expert committee of clinical experts and experts in quality improvement. They are deliberately formulated in broad terms to provide space for the QI teams to specify improvement projects targeted to their local context, within the overall objectives. During QIC implementation, the QI teams engage in national learning sessions about best practices and improvement methods, and they share their experiences of making local changes. Between learning sessions, the teams work with their local QIC improvement projects and monitor their progress. They are supported by local and regional coordinators, such as consultants from quality departments at the hospital/municipal and regional level. The coordinators provide process facilitation and training in QIC methodology as well as data to support progress assessment [27, 41]. The tasks and roles of the coordinators and QI teams are specified on a conceptual and overall level in role descriptions, formulated at the national and in some regions, the regional levels.

Regarding QIC methodology, it was politically decided to apply an adjusted version of the IHI’s Breakthrough Series collaborative methodology as a common framework for all QICs. This includes key components of the Model for Improvement, including Quality Improvement Charter, Driver Diagram and Plan-Do-Study-Act Cycles (PDSA) [7, 42]. Furthermore, it includes the qualitative assessment tool Model for Understanding Success in Quality (MUSIQ) [43] (see Table 1 for an overview of the framework).

**Table 1:** Overview of QIC methods and tools

QIC methods and tools		Description
Model for Improvement	Quality Improvement Charter	A guiding document that helps the QI team structure their improvement project and develop and communicate their shared vision. The charter concretises the improvement project, including the goals and measures, and identifies the members of the improvement team.
	Driver Diagram	A visual display of the QI teams’ theory of what drives or contributes to the achievement of the aim of their local quality improvement project.
	Plan-Do-Study-Act cycles (PDSA)	A four-stage problem-solving model to assist the QI team in implementing and adapting their ideas for change to practice in their specific context.
Model for Understanding Success in Quality (MUSIQ)		A qualitative assessment tool designed to help the QI teams assess aspects of their organizational set-up and local context that may affect the success of their quality improvement project. It provides the opportunity to make adjustments to the project and support systems during the implementation period.

### Case selection

We included as sub-cases two QICs: ‘QIC on diabetes in children’ (QIC diabetes) and ‘QIC on upper femur hip fractures among 65 + aged patients’ (QIC fractures). This choice was based on a diverse case selection strategy to allow for the exploration of characteristics associated with the QICs that could be significant for their translation process [44, 45]. The variation primarily related to differences in organizational complexity. For example, the QI teams in QIC diabetes solely included professionals from pediatric hospital wards, whereas QIC fractures was inter-sectoral, comprising teams from both hospitals and municipalities. Additionally, the QI teams’ composition of professionals varied. The teams in QIC diabetes mainly consisted of pediatric doctors and nurses, whereas the QIC fracture teams involved a broader combination of professionals, including doctors, nurses, physiotherapists, occupational therapists, and care assistants from various departments. We expected these QICs to provide different contextual conditions for translation and thus potentially lead to different translation practices and implications of these.

### Data collection

Data included qualitative interviews, participant observation, and documentary material collected from spring 2020 to autumn 2021. Qualitative interviews involved 39 individual and focus group interviews with a total of 98 employees and managers involved in the two QICs at the national, regional, and local level (see Table 2 for an overview of all conducted interviews). The interviews were conducted by video (n = 35) or in- person (n = 4) at a location chosen by the informants^1^. They lasted 45–90 min, and were all conducted by the first author, who is an experienced social scientist and not affiliated with the implementation of the QICs. For all focus groups and individual interviews, a semi-structured interview guide with open-ended questions was used [46]. The interview guide was informed by the theoretical framework and addressed the informants’ role in the implementation process; their use and understanding of QIC methodology; their definition and bounding of the QIC content; as well as experienced challenges and possibilities of the QIC approach. All interviews were digitally recorded, transcribed verbatim, and anonymized.

**Table 2:** Overview of interviews conducted (39 in total)

	QIC Diabetes	QIC Fractures
National project managers	1 (2)*	1 (1)
Regional coordinators	5 (5)	5 (6)
Local coordinators	5 (9)	- **
Expert faculty	1 (3)	1 (5)
Expert faculty chairman	1 (1)	1 (1)
QI teams	8 (22)	10 (43)
In total	21 (42)	18 (56)

* Number of interviews (number of participants)

** Local coordinators in QIC Fractures participated in the focus groups with the QI teams

Participant observations included participation in 32 meetings (approx. 60 h) central to the national, regional and local QIC implementation (e.g. national learning sessions, expert faculty meetings, regional network meetings, and local improvement team meetings). The observations provided insights into translation in practice. Field notes consisted of both descriptive information (factual data) and reflective information (thoughts, ideas, and questions) to support the analysis of the empirical data [47–49].

Finally, we collected documentary material (e.g. meeting minutes and administrative and policy documents) to provide context information regarding the implementation process and central elements of QICs. Furthermore, the documentary material contributed to corroborating and augmenting the interview and observation data [38].

### Data analyses

**Fig. 1:**
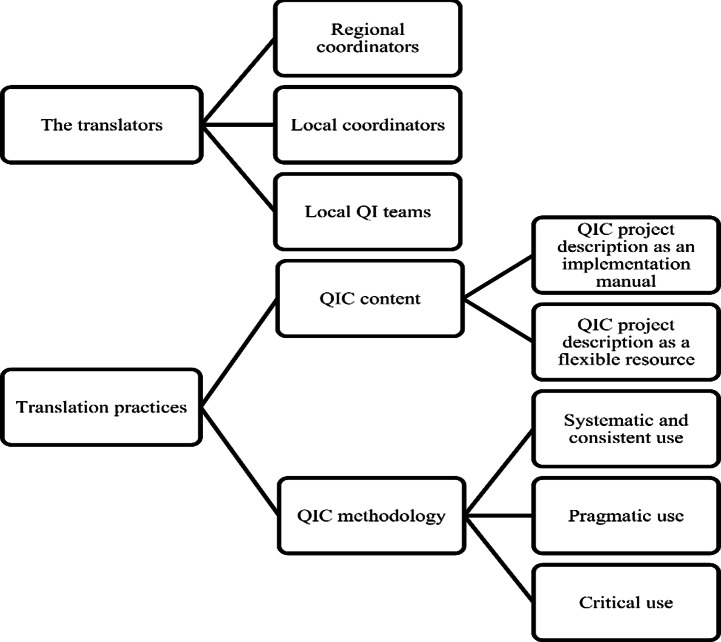
Thematic map

The empirical data was analyzed using reflexive thematic analysis [50, 51]. Informed by the three key aspects of Røvik’s conceptual framework(outlined in the theoretical framework section) and based on a preliminary test-coding and analysis of partial data material, we decided to code our material in three broad theoretical codes focused on (1) translation practices (with three empirical sub-codes: content, methodology, and roles, representing the identified areas of translation); (2) contextual conditions for translation; and (3) implications of translation. All data were coded by the first author using NVivo 12 software (QSR International, Melbourne, Australia). The coded material was then read and analyzed in accordance with Røvik’s conceptual framework. We focused on what aspects of the QICs were translated, which types of translation modes and rules were applied, which rationales for translation were articulated, which and how contextual conditions influenced the translations, and which implications the translations were experienced to have. During this analysis, different clusters of meaning (themes) were identified within each code. As a result, the code ‘translation practices’ was divided into two domains (translators and translation practices), each with a set of themes and in some cases sub-themes capturing different aspects in relation to the domain. The codes ‘contextual conditions’ and ‘implications of translation’ were split up and incorporated into the two domains. Throughout this analysis process, the domains and related themes were investigated in relation to the theoretical framework and the full dataset to ensure validity, and the results were discussed among all authors. The final domains, themes, and sub-themes and their connections are displayed in a thematic map (Fig. 1), and further described at the beginning of the results section below.

### Ethics approval and consent to participate

The study was conducted in accordance with the Declaration of Helsinki and registered in the register of public research projects in the Central Denmark Region (file no. 1-16-02-285-19). The Central Denmark Region Committees on Health Research Ethics deemed our study not to be a health research study, according to the Consolidation Act on Research Ethics Review of Health Research Projects, Consolidation Act number 1083 of 15 September 2017 Sect. 14, and thus, does not require consideration by an ethics committee. Before all observations, written and oral information about the study was provided to all participants and oral, informed consent was obtained. For the interviews, all participants were thoroughly informed about the study and informed, and written consent was obtained. In the analysis and presentation of the collected data, all participating QI teams and individual participants were anonymized.

## Results

In accordance with the structure of the thematic map (presented in Fig. 1), the analysis falls in two parts. In the first part, the analysis of the domain ‘The translators’, we analyze three main groups of translators involved in the implementation of the QICs: the regional coordinators, local coordinators, and local QI teams, with a focus on the translation of their roles in the implementation process. In the second part of the analysis, the analysis of the domain ‘Translation practices’, we analyze how the translators translated two key components of the QICs: the content and the methodology. In relation to both parts of the analysis, we focused on identifying different copying, modifying, and radical practices of translation and their underlying rationales. Furthermore, we focused on how translation practices were influenced by different contextual conditions.

### The translators

#### Regional coordinators

In both QICs, the regional coordinators shared an understanding of their role in QIC implementation, which largely corresponded to the formal role description provided at the national and regional levels before implementation. Thus, all regional coordinators perceived themselves as regional process facilitators, and core tasks involved coordination and facilitation of regional QIC activities; support to local coordinators; facilitation of knowledge sharing among local QI teams; and provision of linkage between the national and regional level of the QICs. Several coordinators articulated the role as a responsibility for providing necessary regional infrastructure:

I think of it as providing infrastructure. We help in keeping the regional implementation on track and running as it is supposed to; that people locally and regionally have the necessary support to be able to do their improvement work. (Regional coordinator, QIC diabetes)

Despite the shared understanding, the regional coordinators translated the role descriptions differently.

Most regional coordinators replicated the formal role description and generally did not engage in direct support of the QI teams, as they perceived this task to be within the local coordinators’ jurisdiction. However, a few regional coordinators modified their role to include the provision of occasional or continuous direct support of the local QI teams, despite it not being part of the formal role description. They did so by, for example, participating in local QIC meetings; providing methodological support; acting as a driving force; and acting as the go-to person in cases of implementation challenges. Contribution to the definition and delimitation of QIC content was, however, not considered part of the support. One regional coordinator explained her direct support as a non-deliberate sliding of tasks among her and the local coordinators, while another coordinator explained that a local coordinator had quit and not been replaced yet. Thus, in both cases, translation was made with reference to local coordinators’ engagement and role enactment, indicating an interrelatedness among the actors’ translation practices. A third regional coordinator performed direct support because her manager encouraged regional coordinators to offer consultancy support for local quality improvement work:

It has become this way because my boss really wants us to provide quality improvement consultancy support, as we have an improvement unit in our office that needs to be put into play. (Regional coordinator, QIC fractures)

Thus, in this case, translation was a strategic managerial decision. Furthermore, the analysis showed that for several regional coordinators, the role translation was made with reference to an experienced high level of transformability in the formal role descriptions that allowed for co-determination of the role.

#### Local coordinators

Across the QICs, the local coordinators’ understanding of their role in QIC implementation largely corresponded to the formal role description they were provided with before implementation. Thus, they perceived themselves as facilitators of the local QIC work performed by the QI teams, both in terms of process facilitation and methodological support. Core tasks involved facilitating local QIC meetings; providing data support; facilitating understanding and use of the QIC methodology; and acting as a link between the regional and local QIC levels. None of the coordinators perceived the definition of the QIC content as part of their tasks. Despite this shared understanding, the coordinators translated the role descriptions differently, particularly in terms of the degree of active involvement in the QI team’s work. As with the regional coordinators, a clear motivator for this translation was an experienced high level of transformability in the formal role description. The analysis showed a continuum of active involvement containing three main types: staying on the side-line; acting as driving force; and being a front-runner.

‘*Staying on the side-line*’ characterized a role much in line with the role description, oriented towards ensuring that the members of the QI team understood their tasks and could lead the process themselves, as the following quote exemplifies:

To me, supporting the local team is about handing over the implementation task to them in a good and understandable way, so that it makes sense for them, they know what to do and they become comfortable with leading the process themselves. (Local coordinator, QIC diabetes)

Furthermore, the coordinators supported the team’s understanding of and work with the QIC methodology. Finally, they disseminated information from regional and national QIC fora and ensured that the information was understood by the QI team. The coordinators substantiated their role enactment with a deliberate desire to make the QI teams take responsibility for their QIC project and act as the main driver of their implementation process. Furthermore, for a few coordinators it was also a pragmatic matter of available time and resources.

*Acting as a driving force* characterized most of the local coordinators’ role translation and included more active involvement in the team’s work. In terms of process facilitation, they kept track of the timeline; ensured that meetings were held and followed up on; ensured that QIC methodology was applied; ensured data measurement and monitoring; and ensured a continuous focus on the QIC. One coordinator described the role as follows:

For me, supporting the team is about being a driving force on the team, one that keeps track of the process and the team’s improvement work. For example, how often do we meet? How are things going? Have we used the QIC tools properly? Could we do something more or else? There is a risk that the team will lose such focus sometimes, as the members also have clinical tasks to take care of. (Local coordinator, QIC diabetes)

In terms of methodological support, this group of coordinators also took on a more active role and engaged in a larger degree of modification of the formal role description. For example, one coordinator undertook the writing tasks as the team members felt too busy to do it themselves. The main rationale behind the active engagement was a sense of responsibility for getting the QI teams successfully through the implementation. Furthermore, they felt a responsibility for ensuring that the team made proper use of the QIC methodology, as they perceived this important for the outcomes of the QIC.

Finally, *being a front-runner* characterized few local coordinators’ role enactments. It involved a more radical translation of the formal role description, as these coordinators not only supported the QI teams but also made decisions on their behalf. For example, one coordinator critically assessed and decided on behalf of the team which parts of the QIC methodology to use and which not to use:

The MUSIQ score we don’t use because I’ve assessed that it has no value for our work. I think it’s rubbish, so why spend precious time on it? I know I become some kind of team leader when I make such assessment, but I do it to spare the team and because I think they should spend their time on more valuable tasks. (Local coordinator, QIC fractures)

Similarly, a few coordinators critically sorted which regional and national information to pass on to the team and how to communicate it. As evident from the quote, the coordinators primarily substantiated this role enactment with deliberate considerations of the appropriate use of the QI team’s limited resources. In any case, this role enactment provided different contextual circumstances for the implementation and translation of the QIC at the QI team level.

The analysis showed differences in the ways coordinators enacted their roles according to the specific QIC. For example, several coordinators enacted a more active and facilitating role in QIC fractures that covered multiple departments and sectors, as the need was perceived as larger:

Actually, I think I have a lot of freedom to decide how much to support the team and how to do so. However, sometimes I have to support the team more actively than others; for example, when the QIC is inter-sectoral or there are many departments involved, such as in QIC fractures. In these cases, I have to take on a larger and more actively facilitating role to ensure cohesion and progress in the implementation process. (Local coordinator, QIC fractures)

This reasoning suggests the significance of the organizational complexity of the QIC for the performed translation practices. Furthermore, the analysis showed that several local coordinators continuously adjusted their role enactment in response to the local QI teams and the regional coordinators, suggesting an interrelatedness between the translation practices performed by different actors. For example, several coordinators described how they regulated their level of involvement depending on the engagement, resources, and competencies of the local team.

#### Local QI teams

All local QI teams shared an understanding of their role in QIC implementation that corresponded to the formal role description provided. Thus, all QI teams understood themselves as having the practical responsibility for the local implementation. Core tasks included turning the QIC content into local goals, measures, and change initiatives; implementing change initiatives through the use of QIC methodology; and collecting data to measure the impact of the changes.

There were, however, small differences in the way the local QI teams translated and enacted their role. This particularly related to the extent to which they understood themselves as active actors in the implementation process, something that was not stated clearly in the formal role description. Most teams saw themselves as active agents with responsibility for both development and definition and implementation and application of QIC content and methodology. They substantiated this with a perceived high level of transformability inherent in the QICs, which allowed a large degree of freedom to co- or self-determine the QIC content:

I think we have complete freedom to define our content. Of course, we have taken into consideration the goals addressed in the project description, but we have worked with what we found important in our context. (Local team, QIC diabetes)

For most of these teams, this leeway also applied to the QIC methodology. A few teams enacted a less active role that included simply carrying out what had been decided at the national and regional levels. In contrast to the more active QI teams, they experienced a low level of transformability in the QICs. Thus, they perceived the QIC content as given by the QIC project description, and not as something they should contribute to defining and developing:

Well, the different elements of the improvement initiatives were given by the project description, so we really just had to implement them. That can be difficult enough, but we have really just had to take the project description and implement the elements step by step. (Local team, QIC fractures)

Also, they experienced the QIC content as highly meaningful in their local context.

### Translation practices

#### QIC content

As mentioned, the QIC content, including focus areas, goals, and measures, was formally defined on the national level. In both QICs, it was mandatory for the QI teams to work with all focus areas and measures. However, the QI teams were also expected to develop local improvement projects and formulate local goals within the project framework. Thus, the QIC content encompassed transformability that made translation an inherent part of the QIC implementation. As argued in the first part of the analysis, the translations mainly took place at the level of the local QI teams.

The analysis identified two types of translation: QIC project description as an implementation manual; and QIC project description as a flexible resource (see Table 3 for an overview of the two types of translations).

**Table 3:** Summary of applied translation practices in translation of QIC content and methodology

	Translation practices
**QIC content**	*QIC project description as an implementation manual* − Copying strategy involving intentional effort to implement the QIC focus areas and measures one to one
	*QIC project description as a flexible resource* − Starting with low-hanging fruit; modification strategy involving a deliberate choice of focus areas, measures and initiatives that promised results without requiring extensive resources − Continuing along a track already initiated; modification strategy involving a deliberate choice to focus on parts of the QIC content that were also in focus in other local improvement projects − Demonstrating clear results; modification strategy involving a deliberate choice to focus on QIC content that was not already covered by existing projects − Complying with organizational needs; modification strategy in which practices of selecting and prioritizing QIC content were substantiated by organizational considerations and needs
**QIC methodology**	*Systematic and consistent use* − Copying strategy involving an intentional effort to apply the QIC methodology in a systematic and consistent way
	*Pragmatic use* − Informal use of the model for improvement; modification strategy involving that the principles of the model for improvement were applied, but where not all formal requirements and procedures were strictly followed − Integration of QIC methodology with existing quality improvement methodology; modification strategy involving that the QIC methodology was combined and aligned with quality improvement tools and methods already used in the recipient context
	*Critical use* − Radical translation strategy involving practices of intentional omission of parts of the QIC methodology

##### QIC project description as an implementation manual

Treating the QIC project description as an implementation manual was a translation practice performed by only a few QI teams, which made an intentional effort to implement the focus areas and measures one to one. As such, the translation performed by these teams could be understood as attempts of replication, reflecting an understanding of the QIC project description as an obligatory manual to follow, encompassing a very low level of or no transformability. Furthermore, the analysis showed that the replicating QI teams found the QIC content highly valuable and applicable in their local context. Thus, the replicating translation practices were a meaningful, intentional local practice.

##### QIC project description as a flexible resource

The main group of QI teams treated the QIC project description as a flexible resource. This involved translation practices of selection and prioritization, i.e., different modifications that involved toning down or leaving out certain aspects of the QIC content in order to concentrate on other aspects. The analysis identified four main types of modification strategies used in the translation process: Starting with low-hanging fruit; continuing along a track already initiated; demonstrating clear results; and complying with organizational needs.

*Starting with low-hanging fruit* was a pragmatic strategy that involved a deliberate choice of focus areas, measures, and initiatives that promised results without requiring extensive resources. For example, some teams in the inter-sectoral QIC fractures began with initiatives that involved only one department or sector, as implementation was considered easier when not depending on others. This finding also points to the significance of the organizational complexity of the QIC for the translation practices performed. For other teams, the strategy of low-hanging fruit involved starting with initiatives considered to be the easiest to implement, even though starting with more difficult initiatives could potentially lead to greater results:

We decided to approach the project by saying what will be the easiest way to start instead of beginning with the hardest parts, though these may have been where we could achieve the largest improvement. (Local team, QIC diabetes)

Central to this choice were considerations of available resources and the timing of the QIC. Thus, several teams expressed how the mandatory participation in the QIC and lack of influence regarding the timing of the QIC forced them to engage in such modifications because they were short on resources and involved in multiple other improvement and research projects at the time of the QIC.

*Continuing along a track already initiated* was a strategy that involved a deliberate choice to focus on parts of the QIC content that were also in focus in other local improvement projects. This strategy exemplifies that modification practices are often contingent on factors in the recipient context. A clear rationale behind this strategy was the possibility of using the QIC as a catalyst for the existing improvement projects. To other teams, it was considered motivating and less resource intensive to continue along a track that was already initiated:

When we got the project description, we discovered that some of the recommended change initiatives were things that we had already made progress on. That whetted our appetite, to see that we were moving in the right direction and had good experience to draw on. I think that makes the QIC work more exciting and easier to handle. (Local team, QIC fractures)

*Demonstrating clear results* was a third modification strategy where existing improvement projects were also used as a frame of reference, however differently. Thus, the strategy involved a deliberate choice to focus on QIC content that *was not* already covered by existing projects. A main rationale for this was a strategic intention to achieve the largest results in areas where improvement initiatives were not already taking place. For others, the underlying rationale was to be able to distinguish between the results of different coexisting projects to determine their individual effects:

As we were already doing a care transition study at the diabetes center, we chose to exclude that part and focus on the others. It would be too hard to tell what was working in the two projects and what was responsible for which effects if we did both projects at the same time (Local QI team, QIC diabetes)

*Complying with organizational needs* was a final modification strategy in which practices of selecting and prioritizing were substantiated by organizational considerations and needs. For example, some teams focused on QIC content that was considered strategically important by either local or regional management. Others prioritized areas where the department performed poorly or wanted to improve the most. Finally, some teams adjusted the QIC content to address the local organizational context and needs. In QIC fractures, some municipal teams, for example, broadened their local improvement project to cover a larger target group to make the QIC more relevant in the municipal context:

In the QIC, we have chosen to focus on delirium as a complication. That is something we see among many patients other than those with hip fractures. So, we actually decided to let this QIC initiative become part of our assessment of other patient groups that we receive from the hospital. This decision has made the QIC much more relevant for us. (Local team, QIC fractures)

Similarly, a few QI teams prioritized focus on change initiatives that could help solve existing organizational needs, for example, upgrade of qualifications among newly appointed nurses. This testifies to the significance of the local contextual features in local translation processes. Furthermore, it points to a more general concern when implementing new organizational ideas; that to be considered relevant and engaging, they have to fit with local strategies and needs, regardless of whether this implies modifications of core elements of the idea.

#### QIC methodology

As mentioned, it was politically decided to apply an adjusted version of the IHI’s Breakthrough Series collaborative methodology as a common framework. In both QICs, it was strongly recommended to apply the framework in the regional and local QIC implementation. However, the QIC methodology had a certain level of transformability, in terms of methodological latitude to apply additional methods and tools in appreciation of existing, regional, and local improvement competencies. This transformability gave rise to translation of QIC methodology, in terms of varying degrees and types of use of the different tools and methods, among regional coordinators, local coordinators, and the QI teams.

Three overall types of QIC methodology translation practices were identified: systematic and consistent use; pragmatic use; and critical use of QIC methodology (see Table 3 for an overview of the three types of translations).

##### Systematic and consistent use

Systematic and consistent use of QIC methodology could be conceptualized as a replicating translation strategy based on practices of copying. This type of translation was only found in a few QI teams, who understood the QIC methodology as mandatory and necessary for successful implementation. To these teams, the QIC methodology encompassed a very low level of or no transformability. Importantly, the teams also reported the methodology to be clinically meaningful. For example, a team in QIC fractures found the Model for Improvement helpful in concretizing their improvement project and measurement practices:

We apply the model for improvement quite systematically. It’s a really nice tool to focus our improvement project with, both in terms of what we want to improve and how we will do it. It just makes it much more concrete and manageable. (Local QI team, QIC fractures)

Thus, the replicating translation strategy came out as a meaningful and intentional practice.

##### Pragmatic use

Pragmatic use of QIC methodology was the most common type of translation and could be conceptualized as a modification strategy. It involved various adjustments including additions of and integration with own methods and tools, and omission of QIC methods and tools. Two types of pragmatic use were particularly present: informal use of the Model for Improvement; and integration of QIC methodology with existing quality improvement methodology.

*Informal use of the model for improvement* was a modification strategy where the principles of the model were applied, but not all formal requirements and procedures were strictly followed. Many teams expressed such an approach in relation to the driver diagram and PDSA methodology, as the following quote illustrates:

We haven’t worked systematically with the templates for the driver charter and the PDSA cycles. However, we have followed the principles of the Model for Improvement in practice. Thus, initiatives and ideas have continuously been born, tested, and implemented, but without any formal completion of the PDSA cycles and driver charter. (Local team, QIC diabetes)

Thus, these teams did not systematically collect and assess data to refine the changes, and they did not always document their process in the formal templates. A main rationale for this was that a more systematic use was not considered essential for the QIC outcomes. Thus, several QI teams highlighted the importance of following the method in practice, rather than documenting and systematically monitoring its use. For some QI teams, this led local coordinators to take on the monitoring and documentation task, as they considered it important for the outcome of the improvement project. Other reasons for informal use included the lack of competencies in the QI team and finding it difficult and time-consuming to fully integrate the systematics into everyday practice.

*Integration of QIC methodology with existing quality improvement methodology* implied that the QIC methodology was combined and aligned with quality improvement tools and methods already used in the recipient context. In two regions, this integration was a deliberate, strategic decision made on the regional level and supported by the regional coordinators. In other cases, integration was decided at the local level and mainly performed by the local coordinators. As evident from the quote below, a key rationale for integrating methodologies was a deliberate attempt to establish methodological recognizability and avoid conceptual confusion in the QI teams:

We have chosen to try to integrate the QIC methods with the methods of our existing regional quality improvement approach, and speak that language more than the QIC language, to avoid confusing the clinicians who are used to working with our approach. (Local coordinator, QIC diabetes)

Central to this rationale was also a wish to provide the best possible conditions for the institutionalization of the QIC methodology in the participating organizations and for creating engagement among professionals, a consideration relevant and applicable to the implementation of organizational ideas more generally. Another rationale was that existing methods and tools were perceived to support and add value to the QIC methodology and thus to the QIC implementation and outcomes. Finally, a third set of rationales were more pragmatic and concerned use of resources and existing local competencies.

##### Critical use

The third type of translation was critical use of QIC methodology, which could be conceptualized as materialization of radical translation. There were only a few examples of critical use, and they came out highly intentional. They involved practices of omitting parts of the QIC methodology. Particularly, the qualitative assessment tool MUSIQ was subject to omission, but in some cases, other tools and methods were also deliberately omitted.

Critical use was substantiated by an assessment of the perceived added value of the methods compared with the resources needed to learn and apply them:

We critically assess which of the methods are meaningful to us to work with. (…) If we were to spend time on all the more abstract tools such as MUSIQ, then we would still be sitting around trying to figure out how to do it and what to address. (Local QI team, QIC fractures)

In most cases, such reasoning took place among the QI teams’ professionals. However, on one team, the local coordinator decided to omit selected methods on behalf of the QI team.

Other rationales were more pragmatic. For example, some teams chose to apply methods and tools already in use in their local context or known by team members, as was the case in the following example:

We haven’t used the PDSA cycles. Our colleague, who has implemented our initiative on mouth hygiene, has another quality improvement toolbox because of her education, so she uses that one. And we have that freedom, though we have committed ourselves to the Model for Improvement. (Local team, QIC fractures)

Where critical use in a few QI teams resulted in no (or close to no) use of QIC methodology, other teams used several of the QIC methods and tools. These QI teams and the local coordinator perceived the QIC methodology as a “buffet” from which they could pick and choose, or as encompassing a very high level of transformability. The performance of such radical translations draws attention to the theoretical notion of appropriate and less appropriate translation practices and raises the question of how radical translations can be before they are no longer accepted as translations and before they wash out the core – and here evidence-based – elements of the intervention, formally chosen because of perceived importance for the intervention outcomes.

## Discussion

Based on a qualitative case study of the use of QICs within a Danish national program for quality in healthcare and drawing on Scandinavian institutionalist translation theory, this study shows how translation is an inherent part of QIC implementation. The QICs were not implemented in a linear way across organizational levels and contexts. Instead, they were adjusted through complex practices of replication, modification, and alteration to make them more appropriate in the specific contexts. Translation theory offers a perspective and a conceptual language to describe this translation process that comes along with the implementation of QICs. More specifically, translation theory made it possible to analyse the patterns of translations performed by involved implementers, that is, their applied translation strategies and practices, as well as the contextual conditions that influence these. Such a perspective, we believe, is a valuable key to obtaining a deeper understanding of how QIC implementation unfolds differently in different local contexts, which provides a better foundation for assessing the outcomes of QIC implementation that may contribute to promoting more effective QICs to improve quality in healthcare.

### The dynamics of translation

Returning to our proposed contribution of providing insights into the dynamics of the translation processes inherent in QIC implementation, our findings contribute with at least three important insights.

First, our findings suggest that the translation processes particularly involve the translation of key implementers’ role descriptions, the QIC content, and QIC methodology. In the translation of all three components, the translators drew on different translation strategies and practices in which they copied, omitted, added to, altered, and mixed the QIC components with elements from their own local and regional contexts to different extents. The translations of all three components mainly materialized as kinds of modification, while there were fewer examples of copying and radical practices; the latter were only present in relation to role descriptions and QIC methodology. Our findings confirm the results of implementation studies within health services in general [21, 32, 35, 52] and the few existing QIC implementation studies, which have stated that QICs are not implemented linearly, but shaped by various translations and depended on local negotiations [9, 17, 21]. However, our identification of the specific translation strategies and practices applied contribute to the existing QIC literature with a more detailed understanding of the translation processes embedded in the implementation of QICs in local contexts. Furthermore, our findings support the theoretical claim made by Røvik (2016) [25] that translation is a rule-based activity guided by underlying modes and rules of translation. We, therefore, propose that Røvik’s theoretical concepts provide a valuable framework to reflect on implementation practices and to consider whether a given practice is appropriate to obtain the desired effects.

Second, our findings point to the importance of context for translation. Regardless of the specific strategies and practices applied, a key rationale for translation was to make the QIC relevant, meaningful, and manageable in the local or regional context. Particularly, our findings suggest five features of the recipient context that influence the translations performed: previous and concurrent improvement projects; existing methods and tools for quality improvement; available resources, time, and competencies; and management priorities and organizational needs. In addition to these features, our findings demonstrate the importance of the transformability of the QICs as a contextual condition. Both QIC role descriptions, methodology, and content formally encompassed leeway for local and regional adjustments that the implementers positively evaluated and took advantage of in defining and implementing the QIC components. Finally, in addition to the features of the recipient context and the transformability of the QICs, our findings indicate that the level of organizational complexity may be a third contextual condition influencing the translation strategies and practices performed. Thus, in QIC fractures, several actors performed translation practices in direct response to the large complexity of the QIC in terms of its inter-sectoral and multi-professional organization; for example, enacting a more active and facilitating role as local coordinator or focus on change initiatives that only involved the QI team’s own department or sector. The plurality of contextual conditions influencing the translation practices involved in QIC implementation, point to how context cannot be understood uniformly, but is to be understood as a multidimensional, multifaceted configuration of factors [53]. Furthermore, the empirical findings show that the translation practices are influenced by different contextual conditions at the same time, which in accordance with Røvik (2016) suggests that context exerts influence through organization-specific combinations of factors. Also, our findings suggest that context is not solely a backdrop to action influencing translation practices, but also an actively applied element in the justification of translations made [25, 53]. In the implementation of QICs, these findings thus suggest the importance of paying attention to the significant and complex influence of context on translation practices. Our findings point to the particular significance of features of the recipient contexts, and the transferability and organizational complexity of the QICs. We encourage future research to further examine the significance and complexity of context. Particularly, it would be relevant to further examine the significance of the translatability of the QICs and the degree of similarity between the source and recipient contexts.

Third and finally, our findings demonstrate an interrelatedness of the translations performed by the different actors involved in the QIC implementation. The translations performed at one level of the QIC became an important precondition and frame for the translations performed at the other levels. Similar interrelatedness has been identified by other translation scholars within healthcare and other sectors (e.g. [16, 54]). Nielsen et al. (2021), for instance, found that ideas about mobile technology use for caregivers travelled in multiple directions as the adopting homecare organizations and other key actors engaged in diverse translation practices, and that these multiple translations together shaped the morphing of the ideas. Our study contributes with a focus on analyzing interrelatedness within a case where the actors are involved in a joined implementation process and thus interdependent. Our study clearly shows that translation made by one actor influences the space for translation action for other actors involved in the same implementation activities. We suggest paying attention to the interrelatedness of translations, as it contributes important knowledge about the dynamics of translations. In practice situations, this means that translation decisions made early in the process set a defining framework for implementation opportunities in later stages.

### The experienced implications of translation

A key assumption within translation theory is that translation makes a difference. That is, translation has implications for the shaping and outcomes of the idea under implementation [25, 26, 32–35]. The empirical literature addressing such implications is, however, sparse. In responding to our second proposed contribution of discussing the experienced implications of the translation processes, we add to this literature by arguing that the translation of QICs has at least two important implications.

First, our findings point to the transformative power of translation. The various translations performed during the QIC implementation led to a transformation of the QICs into various regional and local versions. Depending on the translation strategies and practices applied, these versions are characterized by heterogeneity rather than homogeneity. A similar transformation process is identified by Andersen and Røvik (2021) [32] in relation to the implementation of lean. They argue that such transformation may explain the challenges of measuring the effects of the intervention. We suggest that the same may apply to QICs. Because of the translation processes embedded in QIC implementation, QICs are not just QICs but are numerous materialized versions of the original QIC. Such complexity may be difficult to account for in effects studies and thus may explain the varying evidence of QIC effects [3–6, 32]. Therefore, we encourage future research to examine the relations between local translation processes embedded in QIC implementation and the effects of the QICs.

Second, our findings suggest that translation may be significant for the outcomes of the implementation process and the QIC intervention in two important ways. On the one hand, translation may positively contribute to the organizational institutionalization of the QICs and encourage engagement among the professionals involved. Among regional and local coordinators, these two goals were key rationales for the translation of QIC content and methodology. Furthermore, several local QI teams highlighted the QIC transformability as positive and engaging by providing a leeway to create local QIC projects tailored to specific local needs and resources. In their study of the transformation of ‘interdisciplinary teamwork’ into new everyday professional practices, Reay et al. (2013) [55] observed that successful institutionalization of new practices had the best possibility of occurring in bottom-up processes where professionals designed the initiative themselves. Thus, the transformability inherent in the QICs may – besides engagement among professionals – positively contribute to regional and local institutionalization of the QIC. However, the findings of the present study only allow conclusions on intentions and perceptions as experienced by QIC participants. Thus, we encourage future research to further examine the relationship between translation and creation and sustainment of professional engagement and organizational institutionalization.

On the other hand, translation may influence the effects of the QICs. As suggested by Andersen & Røvik (2021) [32], some translations may lead to successful interventions, whereas others may cause the interventions to fail. Or as suggested by Røvik (2016) [25], it may be possible to identify appropriate and less appropriate translations. The QIC content and the QIC methodology were formulated and chosen based on evidence. However, simultaneously formal leeway to regional and local adjustment was provided with no considerations on how many and which adjustments were acceptable in order to be able to achieve the stated aims and outcome. As clearly evident from our findings, regional and local translators engaged in rich translations, some of which were rather radical, particularly in terms of QIC methodology. As our findings do not allow us to conclude on the relationship between such translations and the achieved effects of the QICs, we propose that future research focus on that relationship. Furthermore, we encourage researchers as well as practitioners planning and working with the implementation of QICs and similar interventions to pay attention to the need for balancing the two main concerns: providing local leeway to adjustment of the QIC to fit the local context, needs, and resources in order to positively contribute to organizational institutionalization of the intervention and professional engagement; and making sure that the core and evidence-based elements of the intervention are not washed out when implementing and translating them in local contexts, leading to potentially negative consequences for their effects. Obtaining a balance between these two concerns is critical for making investments in QICs valuable.

### Methodological considerations

To evaluate the findings of the present study, some methodological considerations must be taken into account. First, we based our analysis on a qualitative case, and therefore the potential for transferring the results to other settings must be considered. However, by providing a detailed description of the QICs alongside ample contextual information, we have aimed to enable readers to meaningfully assess the relevance and applicability of the findings to their context. Second, our study reported on the experienced implications of translation. However, we did not collect quantitative data to assess the actual implications of translation for the outcomes of the QIC implementation process and intervention. The collection of such quantitative data constitutes an important area for future studies. Third, a key strength of the present study is the comprehensible and triangulated data that provided a thorough basis for the analysis. The combination of documents, participant observation, and interviews provided a holistic foundation for gaining insight into the micro-practices of implementing QICs and addressing the research aims.

## Conclusion

The present study investigated the dynamics of the translation processes inherent in QIC implementation, and the implications of the translation processes as experienced by the participating actors. Our study demonstrates that key actors at different organizational levels engaged in translation of their implementation roles, and the QIC content and methodology. They drew on different translation strategies and practices that mainly materialized as kinds of modification. The translations were motivated by deliberate, strategic, and pragmatic rationales, contingent on combinations of features of the actors’ organizational contexts, and the transformability and organizational complexity of the QICs. The findings point to a transformative power of translation, as different translations of the QICs led to various regional and local QIC versions. Furthermore, the findings indicate that translation affects the outcomes of the implementation process and the QIC intervention. Thus, translation may simultaneously affect the institutionalization of the QICs as well as the creation of professional engagement positively while negatively influencing the QIC effects.

The findings of our study extend the current research concerning the understanding of the dynamics and complexity of the translation processes embedded in the local implementation of QICs, by providing a more detailed knowledge of the various translation strategies and practices applied by participating implementation actors, the underlying rationales and contextual conditions influencing the translations, and their experienced implications. Thus, our findings constitute a valuable contribution to a more sustainable and effective implementation of QICs in healthcare improvement. We encourage researchers as well as practitioners planning and working with the implementation of QICs and similar interventions to acknowledge translation as an embedded part of the implementation process. Following our results, there is a need for detailed attention to central questions such as: what translations are acceptable and which types do we wish to avoid in order to provide the best circumstances for both organizational institutionalization of the intervention and realisation of the expected intervention outcomes?

## Data Availability

The data that support the findings of this study are not publicly available due to the wish of safeguarding the anonymity of the respondents. However, data are available from the corresponding author upon reasonable request, but restrictions may apply to their availability due to anonymity issues.

## List of abbreviations

QIC: Quality improvement collaborative
QI team: Quality improvement team
IHI: Institute for Healthcare Improvement
MUSIQ: Model for Understanding Success in Quality
PDSA: Plan-Do-Study-Act Cycles

## Footnotes

1 As data collection took place during the Covid-19 pandemic, the majority of interviews had to be conducted by video.
